# Parents’ Attitudes and Beliefs Towards Human Papillomavirus Vaccination

**DOI:** 10.3390/vaccines13111085

**Published:** 2025-10-22

**Authors:** Ivana Kotromanovic Simic, Darko Kotromanovic, Nika Lovrincevic Pavlovic, Jelena Kovačević, Marija Olujic, Danijela Nujic, Matea Matic Licanin, Ivon Matić, Jelena Sakic Radetic, Ilijan Tomas, Vlatko Kopic, Ivan Miskulin, Maja Miskulin

**Affiliations:** 1 Faculty of Medicine Osijek, Josip Juraj Strossmayer University of Osijek, 31000 Osijek, Croatia; ivsimic@mefos.hr (I.K.S.); kotromanovic93@gmail.com (D.K.); nika.felicita@gmail.com (N.L.P.); dr.kovacevic.jelena@gmail.com (J.K.); molujic@mefos.hr (M.O.); danijela.nujic@mefos.hr (D.N.); mamatic@mefos.hr (M.M.L.); jelenasr80@gmail.com (J.S.R.); ilijan.tomas@kbco.hr (I.T.); kopicv@gmail.com (V.K.); maja.miskulin@mefos.hr (M.M.); 2 Oncology Clinic, University Hospital Center Osijek, 31000 Osijek, Croatia; 3 Ophthalmology Polyclinic Dr. Balog, 31000 Osijek, Croatia; 4 Institute of Emergency Medicine of the Vukovar-Srijem County, 32100 Vinkovci, Croatia; ivon.matic@live.com; 5 Health Centre of Osijek-Baranja County, 31000 Osijek, Croatia; 6 Department of Maxillofacial and Oral Surgery, University Hospital Center Osijek, 31000 Osijek, Croatia

**Keywords:** attitudes, beliefs, children, epidemiology, human papillomavirus (HPV), HPV, vaccine, parents, prevention, primary, public, health, vaccination

## Abstract

**Background/Objectives**: The Human Papillomavirus (HPV) is one of the most common causes of STIs, posing a significant public health problem. Today, the transmission of HPV can be very effectively prevented, making it important to vaccinate the target population at a young age. The aim of this study was to examine the attitudes and beliefs of parents regarding the HPV vaccine and the HPV vaccination of their child. **Methods**: This cross-sectional study was conducted in Osijek, Croatia, from June 2021 to September 2022 via a self-administered questionnaire and included 215 respondents. **Results**: The results showed that respondents who would vaccinate their child were significantly more likely to be those who work in the healthcare field, who had heard of the term HPV, who had sought information about the HPV vaccine on their own, and who had received information about vaccination from school doctors. The attitude towards vaccination was more negative among respondents who did not intend to vaccinate their child. In predicting the decision not to vaccinate one’s child against HPV, bivariate logistic regression revealed that the probability of non-vaccination increases with working outside the field of healthcare (OR = 4.61) and a negative attitude towards vaccination (OR = 1.46), while the probability of non-vaccination decreases if information was received from a school doctor (OR = 0.46). Furthermore, multivariate logistic regression showed that there is a significant model in predicting non-vaccination against HPV, consisting of two predictors: working outside the healthcare field (OR = 8.15) and a negative attitude towards vaccination (OR = 1.49). **Conclusions**: Given that parents are responsible for making the decision about HPV vaccination, it is necessary to invest additional efforts in educating them about the importance of preventing HPV infections and the benefits of HPV vaccination itself.

## 1. Introduction

Human Papillomavirus (HPV) infection is one of the most common viral infections among the sexually active population [1,2], with its widespread prevalence and easy transmission suggesting that nearly everyone will come into contact with the virus at some point in their lives. Since HPV infection does not always require sexual contact, vaccination of both genders is crucial before potential exposure [2]. For over 90% of HPV-related cancers in women, cervical cancer is the primary type [3]. Globally, cervical cancer is the fourth most common cancer in terms of incidence and the third most common cancer in women in terms of mortality [4].

Despite the existence of effective vaccines and a global World Health Organization (WHO) strategy to eliminate this cancer by 2030 [5], as well as the fact that vaccination is recommended for children and adolescents, parental acceptance of HPV vaccination plays a key role in increasing vaccination rates, which remains a critical public health challenge [6,7,8]. Recent meta-analyses have shown that despite generally good parental support, HPV vaccination rates vary widely, with striking regional differences. The key factors influencing parental attitudes are complex. On a global level, the main reasons for parental hesitation are fear of adverse effects and concerns about vaccine safety [6]. Conversely, a positive attitude of parents is associated with a higher level of knowledge about HPV and the belief that the vaccine protects against various types of cancer with a physician’s recommendation as the most powerful motivator [9]. In some regions, a positive attitude does not necessarily lead to a high vaccination coverage rate. For example, a meta-analysis in China found that less than half of Chinese parents were aware of HPV and the vaccine, and less than 70% were willing to have their children vaccinated [10]. In Iran, a systematic review also found that although parents’ knowledge and awareness of HPV is low, their attitudes are positive and strong [11]. This discrepancy highlights a critical gap between knowledge and attitudes.

Other studies point to specific demographic and behavioral factors. A systematic review and meta-analysis from sub-Saharan Africa found that 73% of parents were willing to vaccinate their daughters, especially younger parents with higher education, better knowledge, and higher income [12]. Parents in Austria and Poland were less willing to have male children vaccinated [13,14], while those who relied on online sources were less willing to have their child vaccinated [13]. In Poland, however, a strong correlation was found between parental education, parental knowledge about HPV and attitudes towards HPV and HPV vaccine [14]. A study in Sweden showed that parents made independent decisions about vaccinating their daughters because they believed the girls were too young to participate in this decision [15]. According to a French study, parents’ beliefs about HPV and vaccination, including their skepticism towards pharmaceutical companies and internet information sources, were key factors contributing to the low vaccination rate [16]. Studies from several European countries, including Germany, Greece, and Italy, collectively found that lack of information and fear of side effects were the biggest barriers to parental uptake of the HPV vaccine, while a doctor’s recommendation was the most important factor in promoting vaccination [17,18,19]. Similarly, a study from Finland detected a fear of adverse effects as the main reason for non-vaccination, despite the generally positive attitude of parents, high awareness of the link between HPV and cervical cancer, and high trust in official sources of information [20]. According to EU4Health project PROTECT-EUROPE, lack of awareness and knowledge, as well as logistical challenges, are the main barriers to HPV vaccination uptake, while open communication between young people, parents, and healthcare professionals is crucial for increasing vaccination rates [21].

All of these findings illustrate the complex interplay of various factors that influence parents’ decision-making process. Considering that studies examining those parental decisions in Croatia are scarce, this study provides an important insight into the current literature. To better understand these challenges and to assess the situation in our local context, this study aimed to investigate the attitudes and beliefs of parents of 8th-grade students in Osijek, Croatia, regarding the HPV vaccine and the vaccination of their children.

## 2. Materials and Methods

### 2.1. Study Design

This cross-sectional study was conducted between June 2021 to September 2022 in Osijek, Croatia.

### 2.2. Study Setting

The study was carried out among parents of eighth-grade students from four primary schools (Elementary School Tenja, Elementary School Dobriša Cesarić, Elementary School August Šenoa, and Elementary School Josipovac). The selected schools were chosen to ensure the representativeness of the sample, as they included two urban schools (Elementary School Dobriša Cesarić and Elementary School August Šenoa) and two suburban schools (Elementary School Tenja and Elementary School Josipovac). Informative parent-teacher meetings were held in June 2021 and September 2022, during which the research team clarified objectives, methods, expected outcomes, and timelines. Prior to the parent–teacher meeting, approval was obtained from the school principal for the participation of the research team members in the meeting for the purpose of conducting the study. During the parent–teacher meeting, parents had the opportunity to ask questions, after which those willing to participate signed the informed consent form and completed the questionnaire.

### 2.3. Participants and Measurements

We examined parents’ attitudes and beliefs toward the HPV vaccine and HPV vaccination for their children, as well as their intention to actually vaccinate their child, using a questionnaire. The inclusion criteria for participation were obtaining consent to participate and having children attending the 8th grade in the participating elementary schools. The only exclusion criterion was the lack of parental consent for participation. The total number of eighth-grade students in the participating schools was 132 in 2020/2021 and 127 in 2021/2022, yielding 259 eligible students (respectively parents) overall. Only one parent or guardian per student was invited to participate, and some declined or were absent during parent–teacher meetings when informed consent was collected. Our study included 215 parents/guardians, representing a response rate of 83.01%. The questionnaire, used for this study, was specifically developed for the purpose of this study.

The first part of the questionnaire included eight questions addressing the sociodemographic characteristics of the parents, followed by a question assessing their prior familiarity with the term HPV. Subsequently, three questions focused on knowledge related to the HPV vaccine and vaccination. The second part of the questionnaire included the adapted Croatian translation of CHIAS scale and two additional questions aimed to examine the respondents’ intention and attitude towards vaccinating their child against HPV, as well as their willingness to leave such a decision to their child. The complete questionnaire is provided in the Supplementary Material.

The Carolina HPV Immunization Attitudes and Beliefs Scale (CHIAS) was originally published in 2010. and developed to examine attitudes and beliefs about HPV vaccination [22]. Although the CHIAS scale is relatively dated and more recent instruments exist (such as the Parental HPV Vaccine Survey (PHPVS) [23], Health Belief Model-based scales [24], HPV Attitudes and Beliefs Scale (HABS) [25], or the Parent HPV Vaccine Misperceptions Scale [26], and some of these as well as older scales and instruments are well described in the systematic review by Allen et al., 2010 [27], it was the only tool both modified and translated into Croatian at the time of our study, making it the only feasible option for our research. Nevertheless, the CHIAS scale remains a relevant and reliable instrument still widely used in recent research [22,28,29,30,31]. The original CHIAS scale consists of 16 statements and questions. For the purposes of this study, a modified Croatian version of the scale was used, from which six original statements were initially removed, since the HPV vaccine is readily available in the Republic of Croatia, and vaccination is completely free for students attending the fifth, sixth, seventh, and eighth grade of elementary school, students of any grade in high school, as well as regular university students. This version of the CHIAS scale demonstrated Cronbach’s alpha of α = 0.76. However, following exploratory factor analysis, three additional items were excluded as they did not align with the Croatian context. The final version of the adapted and modified CHIAS scale, which consists of seven items and was used in this study, exhibited a reliability coefficient (Cronbach’s alpha) of α = 0.86. The modified and adapted Croatian version of the scale used in this research was obtained with written permission from Delač and Korajlija [32]. This final version of the adapted scale consists of seven statements, and respondents were asked to indicate their degree of agreement with each statement on a four-point Likert scale: 1 = “strongly disagree”, 2 = “disagree”, 3 = “agree”, and 4 = “strongly agree”. The minimum score that could be achieved on the scale was seven, while the maximum was twenty-eight, with a higher score indicating a more negative attitude towards vaccination (i.e., “I worry that the HPV vaccine might cause short-term problems like fever or discomfort.”, “I worry that the HPV vaccine is being pushed to make money for drug companies and/or doctors.”). The last two questions in the questionnaire aimed to examine the intention of the respondents to vaccinate their child against HPV, i.e., the intention to leave the decision about vaccination to their child. Specifically, the official invitation forms given to parents offered only two options: “I agree that my child will receive the HPV vaccine” or “I do not agree that my child will receive the HPV vaccine” [33]. For this reason, we mirrored this structure by using dichotomous (“yes/no”) questions regarding (i) willingness to vaccinate (“Would you vaccinate your child against HPV?”) and (ii) willingness to let the child decide about vaccination (“Would you leave the decision about HPV vaccination to your child?”).

### 2.4. Ethical Procedures

The conduct of this study was approved by the Ethics Committee of the Faculty of Medicine Osijek (date of approval: 10 September 2021, Class: 602-04/21-08/7, Reg. No.: 2158-61-07-21-170) and the Teaching Institute of Public Health of Osijek-Baranya County (date of approval: 8 February 2021, Class: 035-01/21-01/14, Reg. No.: 391-21-4).

### 2.5. Statistical Analysis

Categorical data were presented as absolute and relative frequencies. Differences in categorical variables were tested with the χ^2^ test, and when appropriate, with Fisher’s exact test. Continuous data were described using the median and interquartile range limits. Differences in continuous variables between two independent groups were tested with the Mann–Whitney U test, and the Hodges–Lehmann difference in median was reported, along with the corresponding 95% confidence interval. The influence of respondents’ characteristics on the probability of deciding to vaccinate their child against HPV was assessed using logistic regression analysis, both bivariate and multivariate (stepwise method). All values were two-sided. The significance level was set at α = 0.05. Statistical analyses were performed using MedCalc^®^ Statistical Software version 23.1.1. (MedCalc Software Ltd., Brussels, Belgium, 2025) [34].

## 3. Results

The study was conducted on 215 respondents, who were parents, of whom 187 (87%) were women. The median age of the respondents was 43 years, ranging from 33 to 59 years. The number of children per family ranged between two to seven, with a median of two children. In terms of education, 125 respondents (58.1%) had graduated from high school. Regarding employment status, 190 (88.4%) were employed, and 32 (14.9%) worked in the health care sector. A total of 183 (85.1%) respondents were married. With respect to socioeconomic status, 142 (66%) reported an average income, 10 (4.7%) reported a below-average income, and 63 (29.3%) reported an above-average income (Table 1).

In total, 202 (94%) respondents had heard about the term HPV, and 122 (56.7%) of them individually sought information about the HPV vaccine in Croatia. The most common single source of information was internet portals (37.2%). Overall, 170 respondents (79.1%) would vaccinate their child against HPV, while 63 (29.3%) would leave the decision about vaccination to their child (Table 2).

Respondents who indicated they would vaccinate their child against HPV were significantly more likely to be those who work in the health care field (χ^2^ test, *p* = 0.03), who had heard of the term HPV (χ^2^ test, *p* < 0.001), who had individually sought information about the HPV vaccine in Croatia (χ^2^ test, *p* = 0.004), and who had received information about the HPV vaccine from school doctors (χ^2^ test, *p* = 0.02) (Table 3).

There is no difference in leaving the decision about HPV vaccination to the child (χ^2^ test, *p* = 0.06) among parents who would or would not vaccinate their child against HPV. The majority of respondents (70.7%) would not leave the decision about HPV vaccination to their child.

There was no significant difference in respondents’ age (*p* = 0.88) with respect to their decision to vaccinate (Median (Mdn) = 43 (Interquartile range (IQR) = 40–47)) or not vaccinate (Mdn = 43 (IQR = 40–46)) their child against HPV.

Attitudes about vaccination were assessed using the modified, Croatian version of the CHIAS. The distribution of responses to all questionnaire questions is shown in Table 4.

Respondents who answered that they would not vaccinate their child, compared to those who said they would, significantly more often agreed with statements that the HPV vaccine is promoted for pharmaceutical companies to profit financially, that the HPV vaccine can cause long-term health problems, that the HPV vaccine is not safe, that their child is too young to receive a vaccine against a sexually transmitted disease such as HPV, and that the HPV vaccine is quite new, so they want to wait before deciding whether their child should receive it (Mann–Whitney U test, *p* < 0.001). The overall attitude toward HPV vaccination, based on the CHIAS scale analysis (CHIAS score) was more negative among respondents who answered that they would not vaccinate their child (Mann–Whitney U test, *p* < 0.001) (Table 5).

Bivariate and multivariate logistic regression analyses were performed to examine the influence of respondents’ characteristics on their decision to vaccinate their child against HPV. In predicting the decision not to vaccinate one’s child against HPV, bivariate logistic regression showed that the probability of non-vaccination increases with working outside the field of healthcare (OR = 4.61) and having a negative attitude towards vaccination, as measured by the CHIAS score (OR = 1.46), while the probability of non-vaccination decreases if information was received from a school doctor (OR = 0.46). The initial multivariate logistic regression model included 11 variables associated with the outcome in preliminary analyses. Multivariate logistic regression (using the stepwise method) revealed a significant model for predicting non-vaccination against HPV, consisting of two predictors: working outside the field of healthcare (OR = 8.15) and having a negative attitude towards vaccination, as measured by the CHIAS score (OR = 1.49). The model was entirely significant (χ^2^ test = 65.9, *p* < 0.001) and explained between 26% (according to Cox & Snell R^2^) and 41% (according to Nagelkerke R^2^) of the variance in non-vaccination against HPV, correctly classifying 85% of cases (Table 6).

## 4. Discussion

The article examined parents’ attitudes towards HPV vaccination and their willingness to vaccinate children against HPV. The results showed that parents generally reported positive attitudes and beliefs towards HPV vaccination of their children, since 79.1% of them reported intention to vaccinate their child against HPV. This share of willing parents is higher than shares reported in Poland with 59.1% [14], Ethiopia with 74% and Nigeria with 70–79.2%, but lower than shares reported in Indonesia with 96.1% [35], China with 87.6% [36], and Austria with 81.9% [13]. A recent systematic review found a pooled percentage of 59% of parents with positive intention to vaccinate their children against HPV in European countries, with higher acceptance in Nordic countries [37]. Differences in parents’ willingness to vaccinate against HPV may vary across countries due to factors such as the duration of HPV vaccine availability and the presence or absence of national immunization programs that offer the HPV vaccine free of charge. It has been suggested that parents may be less willing to vaccinate their children against HPV if they have to pay for the vaccine [36,38].

More than half of the study respondents actively sought information about the HPV vaccine on their own, demonstrating significant parental interest in their children’s health and preventive measures. The most commonly used sources of information reported by parents regarding the HPV vaccine were the internet, school doctors, who provide HPV vaccination in Croatia, and the media. Research on parents’ knowledge and attitudes towards the HPV vaccine in Croatia is limited. One other study found that the internet and the media were the most frequently used sources of information about the HPV vaccine among Croatian parents [32]. Similar findings have been reported globally. Several studies and systematic reviews found medical doctors or healthcare providers [8,37,39,40] and the internet [8,39] as parents’ primary sources of information concerning the HPV vaccine.

This study established no association between parents’ intention to vaccinate their children against HPV and demographic factors such as parents’ gender, age, level of education, employment status, or socioeconomic status. There is no similar research in Croatia for comparison, but other studies around the world obtained different results. In Greece, parents’ occupation, education and income were not associated with HPV vaccination of children, but older age of parents was associated with HPV vaccination of children [8]. Higher maternal education was found predictive of parents’ willingness to vaccinate their children against HPV in Ethiopia [35]. Higher maternal education and higher income were positively associated with the willingness to vaccinate their children against HPV among Arab Americans [41]. In Poland, higher parents’ education and male gender were associated with the willingness to vaccinate their children against HPV [14]. In China, parental education and income were not associated with the willingness to vaccinate their children against HPV [36]. In Indonesia and Austria, sociodemographic factors were not associated with parents’ intention to vaccinate children against HPV [13,42]. Since results vary across the countries, we can conclude that every country has to take into an account their own situation and react accordingly.

An important factor identified in this study as positively associated with parents’ intention to vaccinate their children against HPV was receiving information from a school doctor, as opposed to other healthcare professionals, pediatricians, or pharmacists, which were not found to be a significant source of information. This finding reflects the organization of the Croatian public healthcare system, where, unlike some other countries, the HPV vaccine and all other vaccines recommended or obligatory for the school-aged children and adolescents, are administered by the school doctor. Since other medical specialists, like family doctors and pediatricians, do not provide vaccination for school-aged children and adolescents in Croatia, they may not be sufficiently informed or encouraged to educate parents about the HPV vaccine. Furthermore, other sources of information, such as a gynaecologist and informational leaflets, were significantly associated with parents’ intention not to vaccinate their children against HPV. Therefore, additional efforts should be made in Croatia to strengthen the role of school doctors in disseminating HPV-related information. School doctors should be further engaged in health promotion strategies and integrated into different health communication platforms to reach a significant number of parents seeking vaccine-related information. This is consistent with the role and education of school and adolescent medicine specialists, who possess the knowledge and skills required for effective health education and promotion among children, adolescents, and their parents. The importance of receiving a recommendation from a medical doctor for HPV vaccine acceptance has been well-established in several previous studies [1,8,9,37,38,40,41,43,44,45,46,47,48]. One interventional study found that a conversation with a healthcare professional reduced anxiety and concerns associated with the HPV vaccine and was associated with increased HPV vaccine acceptance [49].

Parents employed in the healthcare system reported a significantly higher intention to vaccinate their children against HPV. This finding is expected, as healthcare professionals are generally more familiar with health-related topics and likely have more positive attitudes and greater confidence in the healthcare system, given their active role within it. However, a study from Croatia found no significant difference in the intention to vaccinate female children against HPV between healthcare professionals and other parents, although it did report significantly higher willingness among healthcare professionals to vaccinate male children against HPV in comparison to other parents [50]. A similar positive association between being a healthcare worker and the intention to vaccinate children against HPV was observed in Serbia [38]. On the other hand, a study in China found no association between being medical staff and the intention to vaccinate children against HPV [36].

Parents who were familiar with the term HPV reported a significantly higher intention to vaccinate their children against HPV compared to those who were not. This finding is consistent with previous research [35,36]. Similarly, parents who actively sought information about the HPV vaccine on their own were more likely to express an intention to vaccinate their children against HPV than those who did not, suggesting that these parents took the initiative to educate themselves and make informed decisions. Notably, more than half of the study respondents reported actively seeking such information, highlighting the considerable interest parents have in this issue. Therefore, public health authorities and administrators should prioritize making accurate information about HPV vaccination easily available through a variety of channels, platforms, and settings. These should be tailored to the needs and habits of the current generation of parents, who are interested in preventive measures and the health of their children. In this process, efforts should be focused on the most effective and reliable sources of information as reported by parents.

Another important consideration highlighted by previous research is that stated intention to vaccinate does not always correspond to the actual number of children and adolescents being vaccinated in the following period [13,51]. Some studies have shown that only 38–57% of parents who intend to vaccinate ultimately follow through. This emphasizes the importance of addressing barriers between intention and action, which might include reminders, increased vaccine availability, and opportunistic vaccination [13].

The results of this study also showed that the CHIAS score was predictive of parents’ intention to vaccinate their child against HPV, which is consistent with findings from other studies that used the same tool [22,45]. The association between vaccination intention and specific attitudes identified by the CHIAS points to potential targets for the intervention strategies [22]. In this study, the CHIAS results suggested that public health efforts should focus on uncertainty and concerns about potential harms of the vaccine, which are the issues more commonly reported by parents who were unwilling to vaccinate their children against HPV. The most important factors influencing parents’ intention to vaccinate, as identified by the regression models, were employment in a healthcare-related profession, receiving information from a school doctor, and attitudes measured by the CHIAS score. These findings align with previous research, which has shown that a positive attitude towards the HPV vaccine [35,52] and a greater knowledge about HPV [13] are associated with higher parental willingness to vaccinate. On the other hand, a lower HPV vaccination coverage has been linked to relying on the internet or social media and friends or family as a source of information about HPV [8,13], as well as to negative parental attitude towards vaccination [13] and a history of vaccine refusal [52].

Although this study found that 29.3% of parents would allow their children to make an independent decision regarding HPV vaccination, current Croatian legislation does not recognize this possibility. Therefore, parental willingness to vaccinate their children remains the cornerstone of successful preventive measures against HPV infection. It is thus crucial to enhance parents’ knowledge about HPV and positively influence their attitudes towards the HPV vaccine. Previous research in Croatia has shown that adolescents most frequently seek vaccination-related information on the internet, although they identified health care professionals as the most reliable source. Furthermore, the same research also found that the negative attitude towards vaccination was associated with relying on the internet as a primary source of information and having limited knowledge about vaccination [53]. A recent Croatian study among a nationally representative sample of older adolescents and young adults reported only 18.3% of participants vaccinated against HPV and 35.4% of those unvaccinated willing to get HPV vaccine. The study also suggested the need for raising awareness among both parents and adolescents in order to increase HPV vaccination uptake in Croatia and acknowledged the role of specialists in school and adolescent medicine in educating parents about benefits of vaccination [54].

Systematic reviews recommend targeting those factors proven to promote vaccine acceptability in order to optimize vaccine uptake and tailored interventions [37,40]. Given the fact that the most frequently used source of information about HPV reported in this study was the internet, and that the most effective source of information was the school doctor, a promising national strategy could involve combining the two: placing school doctors, who have significant impact on the intention of parents to vaccinate their children against HPV, on the internet and social media. Other countries have already recommended or implemented the use of social media to educate citizens about HPV and raise awareness, with positive outcomes [13]. Furthermore, systematic reviews also found certain communication strategies of healthcare providers to be more efficient in promoting HPV vaccine uptake and recommended that all clinicians who are HPV vaccine providers should be trained in these communication strategies [55].

### Limitations of the Study

This study has several limitations that may impact the validity of the obtained results. As a cross-sectional study, it is not possible to establish causality, but only associations between the observed variables. Furthermore, the inclusion of a relatively small, convenience-based, and non-representative sample of respondents should be considered, as well as the possibility that respondents may have provided socially desirable answers rather than responses that reflect their true opinions and attitudes. The predominance of female respondents (88.2%) also limits the study’s ability to represent fathers’ perspectives. Additionally, the use of a dichotomous measurement of vaccine willingness may constitute an oversimplification, which represents one of the study’s limitations. Moreover, the study did not account for other potentially influential factors, such as prior experiences with vaccination or exposure to media coverage and public health campaigns promoting vaccination. Finally, it is possible that certain dimensions of parental attitudes were not fully captured or analysed by the scale used.

Therefore, further research is needed to confirm and better understand the relationship between parents’ attitudes and beliefs regarding the HPV vaccine and their intention to vaccinate their children against HPV, particularly within the studied population in Croatia.

## 5. Conclusions

Given the fact that the research on HPV vaccination among adolescents and their parents are scarce in Croatia, this study presents a valuable addition to the research corpus. The findings of this study revealed an overall positive parental attitude toward the HPV vaccine and a high willingness among parents to have their children vaccinated, with the internet and school doctors serving as the most common sources of information. Parental intention to vaccinate was significantly linked to employment in a healthcare-related field, receiving information from a school doctor, and higher CHIAS scores. It is essential that national health promotion strategies are adapted to the unique characteristics of each country’s population and directly address parents’ specific attitudes and concerns regarding HPV vaccination. Developing interventions that are both targeted and culturally sensitive will help public health authorities increase parental awareness and willingness to vaccinate. Ultimately, such measures are expected to reduce the prevalence of HPV infection and decrease the morbidity and mortality associated with HPV-related cancers.

## Figures and Tables

**Table 1:** General subject characteristics.

**Sex**	**[n (%)]**
Male	28 (13)
Female	187 (87)
**Level of education**	**[n (%)]**
Elementary school education	2 (0.9)
High school education	125 (58.1)
Undergraduate or graduate education	79 (36.7)
MSc/PhD	9 (4.2)
**Employment status**	**[n (%)]**
Employed	190 (88.4)
Unemployed	13 (6.0)
Housewife	7 (3.3)
In the process of education (student)	1 (0.5)
Retired	4 (1.9)
**Current partnership status**	**[n (%)]**
Married	183 (85.1)
Domestic partnership	14 (6.5)
In a relationship	2 (0.9)
Single	16 (7.4)
Socioeconomic status *	**[n (%)]**
Below average	3 (1.4)
Slightly below average	7 (3.3)
Average	142 (66.0)
Slightly above average	56 (26.0)
Above average	6 (2.8)
Not answered	1 (0.5)
**Work in the field of health care**	**[n (%)]**
Yes	32 (14.9)
No	183 (85.1)

* socioeconomic status is self-assessed.

**Table 2:** Awareness and sources of information about HPV.

	Number (%) of Subjects
**Heard about the term HPV**	202 (94)
**Individually sought information about the HPV vaccine in the Republic of Croatia**	122 (56.7)
**Information about HPV through**	
School doctor	109 (50.7)
Media (TV/radio/newspapers/magazines)	92 (42.8)
Internet portals	58 (27.0)
Other health personnel	42 (19.5)
Forums	27 (12.6)
Family members/friends/colleagues	20 (9.3)
Pediatrician	12 (5.6)
Pharmacists	11 (5.1)
Other	7 (3.3)
State institutions (e.g., CHIF, CIPH)	6 (2.8)
Educational institutions (kindergarten, school, faculty)	2 (0.9)
**The MOST frequently used source of information about HPV**	
Internet portals	80 (37.2)
School doctor	52 (24.2)
Media (TV/radio/newspapers/magazines)	30 (14.0)
Family members/friends/colleagues	15 (7.0)
Other health personnel	11 (5.1)
Educational institutions (kindergarten, school, faculty)	8 (3.7)
State institutions (e.g., CHIF, CIPH)	7 (3.3)
Other	7 (3.3)
Forums	3 (1.4)
Pharmacists	1 (0.5)
Pediatrician	1 (0.5)
**Would you vaccinate your child against HPV?**	170 (79.1)
**Would you leave the HPV vaccination decision up to your child?**	63 (29.3)

**Table 3:** Distribution of respondents according to characteristics in relation to whether they would vaccinate their child against HPV.

	Number (%) of Subjects According to Whether They Would Vaccinate Their Child Against HPV			*p*
	Yes (n = 170)	No (n = 45)	Total (n = 215)	
**Sex**				
Male	20 (11.8)	8 (17.8)	28 (13)	0.29 *
Female	150 (88.2)	37 (82.2)	187 (87)	
**Level of education**				
Elementary school/High school education	103 (60.6)	24 (53.3)	127 (59.1)	0.38 *
Undergraduate or graduate education/MSc/PhD	67 (39.4)	21 (46.7)	88 (40.9)	
**Employment status**				
Employed	152 (89.4)	38 (84.4)	190 (88.4)	0.36 *
Others	18 (10.6)	7 (15.6)	25 (11.6)	
**Work in the field of health care**				
Yes	30 (17.6)	2 (4.4)	32 (14.9)	**0.03** *
No	140 (82.4)	43 (95.6)	183 (85.1)	
**Heard of the HPV**	166 (97.6)	36 (80)	202 (94)	**<0.001** *
**Individually sought information about the HPV vaccine in the Republic of Croatia**	105 (61.8)	17 (37.8)	122 (56.7)	**0.004** *
**Information about HPV through**				
School doctor	93 (54.7)	16 (35.6)	109 (50.7)	**0.02** *
Media (TV/radio/newspapers/magazines)	74 (43.5)	18 (40.0)	92 (42.8)	0.67 *
Internet portals	51 (30.0)	7 (15.6)	58 (27.0)	0.05 *
Other health personnel	36 (21.2)	6 (13.3)	42 (19.5)	0.24 *
Forums	21 (12.4)	6 (13.3)	27 (12.6)	0.86 *
Family members/friends/colleagues	18 (10.6)	2 (4.4)	20 (9.3)	0.26 ^†^
Pediatrician	10 (5.9)	2 (4.4)	12 (5.6)	>0.99 ^†^
Pharmacists	8 (4.7)	3 (6.7)	11 (5.1)	0.70 ^†^
Other	0	7 (15.6)	7 (3.3)	**<0.001 ** ^†^
State institutions (e.g., CHIF, CIPH)	5 (2.9)	1 (2.2)	6 (2.8)	>0.99 ^†^
Educational institutions (kindergarten, school, faculty)	1 (0.6)	1 (2.2)	2 (0.9)	0.38 ^†^

* χ^2^ test; ^†^ Fisher’s exact test; Bold numbers: Results with statistical significance.

**Table 4:** Self-assessment of attitudes about HPV vaccination (CHIAS).

	Number (%) of Subjects				
	Strongly Disagree	Disagree	Agree	Strongly Agree	Total
The HPV vaccine might cause short-term problems, like fever or discomfort	12 (5.6)	49 (22.8)	105 (48.8)	49 (22.8)	215 (100)
The HPV vaccine is being pushed to make money for drug companies	101 (47)	89 (41.4)	20 (9.3)	5 (2.3)	215 (100)
The HPV vaccine might cause lasting health problem	70 (32.6)	111 (51.6)	26 (12.1)	8 (3.7)	215 (100)
If a teenage girl/boy gets the HPV vaccine, she/he may be more likely to have sex	136 (63.3)	64 (29.8)	12 (5.6)	3 (1.4)	215 (100)
I think the HPV vaccine is unsafe	100 (46.5)	84 (39.1)	23 (10.7)	8 (3.7)	215 (100)
My child is too young to get a vaccine for a sexually transmitted infection like HPV	111 (51.6)	62 (28.8)	26 (12.1)	16 (7.4)	215 (100)
The HPV vaccine is quite new, so I want to wait a while before deciding if my child should get it	88 (40.9)	77 (35.8)	39 (18.1)	11 (5.1)	215 (100)

**Table 5:** Differences in self-assessment of attitudes towards HPV vaccination (CHIAS) in relation to the decision to vaccinate their child.

	Median (Interquartile Range) According to Whether They Would Vaccinate Their Child Against HPV		Difference	95% CI (Confidence Interval)	p *
	Yes	No			
**The HPV vaccine might cause short-term problems, like fever or discomfort**	**3 (2–3)**	**3 (3–3)**	0	0–0	0.77
The HPV vaccine is being pushed to make money for drug companies	1 (1–2)	2 (2–3)	1	0–1	**<0.001**
The HPV vaccine might cause lasting health problem	1 (1–2)	2 (2–3)	1	0–1	**<0.001**
If a teenage girl/boy gets the HPV vaccine, she/he may be more likely to have sex	1 (1–2)	1 (1–2)	0	0–0	0.22
I think the HPV vaccine is unsafe	1 (1–2)	3 (2–3)	1	1–1	**<0.001**
My child is too young to get a vaccine for a sexually transmitted infection like HPV	1 (1–2)	3 (2–4)	2	1–2	**<0.001**
The HPV vaccine is quite new, so I want to wait a while before deciding if my child should get it	2 (1–2)	3 (2–3)	1	1–1	**<0.001**
**CHIAS score total**	12 (10–14)	17 (14–21)	5	4–6	**<0.001**

* Mann–Whitney U test; Bold numbers: Results with statistical significance.

**Table 6:** Prediction of the probability of a negative decision to vaccinate children against HPV (bivariate and multivariate logistic regression).

Do Not Intend to Vaccinate Their Children Against HPV	β	Wald	*p*	Odds Ratio (OR)	95% CI (Confidence Interval)
**Bivariate logistic regression**					
Age	−0.01	0.003	0.96	0.99	0.93–1.07
* Sex (F)	−0.48	1.12	0.29	0.62	0.25–1.51
† Level of education (ISCED 4,6,7 vs. others)	0.30	0.77	0.38	1.35	0.69–2.61
§ Employment status (others vs. employed)	0.44	0.84	0.36	1.56	0.61–3.99
ǁ Work outside of the field of health care	1.53	4.14	**0.04**	4.61	1.06–20.1
** Information about HPV through:					
School doctor	−0.78	5.1	**0.02**	0.46	0.23–0.90
Media (TV/radio/newspapers/magazines)	−0.15	0.8	0.67	0.86	0.44–1.68
Internet portals	−0.84	3.62	0.06	0.42	0.18–1.03
Other health personnel	−0.56	1.37	0.24	0.57	0.22–1.45
Forums	0.09	0.03	0.86	1.09	0.41–2.89
CHIAS score	0.37	37.6	**<0.001**	1.46	1.29–1.65
**Multivariate logistic regression**					
ǁ Work outside of the field of health care	2.09	5.33	**0.02**	8.15	1.37–48.32
CHIAS score	0.40	36.3	**<0.001**	1.49	1.31–1.70
Constant	−6.9	42.3	**<0.001**		

β—regression coefficient. Reference categories: * male; † elementary/high school education; § employed; ǁ work in the field of health care; ** information about HPV through: yes (vs. no). Bold numbers: Results with statistical significance.

## Data Availability

All data are available and can be delivered to anyone upon request.

## Supplementary Materials

The following supporting information can be downloaded at: https://www.mdpi.com/article/10.3390/vaccines13111085/s1, questionnaire.
