# Supplementary material for: Parents’ Attitudes and Beliefs Towards Human Papillomavirus Vaccination
Source: Vaccines (Basel). 2025 Oct 22;13(11):1085. doi: 10.3390/vaccines13111085 (PMC12656538; doi:10.3390/vaccines13111085)
Supplement: Supplementary file 1 [file vaccines-13-01085-s001.zip › vaccines-3885568-supplementary.pdf]

At the beginning, we kindly ask you to answer a few general questions about yourself:

1. How old are you? (e.g., 40 years)

____

2. What is your gender?
a) Male
b) Female

3. Highest level of education completed:
a) Elementary school education
b) High school education
c) Undergraduate or graduate education
d) MSc/PhD

4. Employment status:
a) Employed
b) Unemployed
c) Housewife
d) In the process of education (student)
e) Retired

5. Please assess the socioeconomic status of your family:
a) Below average
b) Slightly below average
c) Average
d) Slightly above average
e) Above average

6. Do you or any of your immediate family members work in the field of health care?
a) Yes
b) No
c) I don't know

7. What is your current relationship status?
a) Married
b) Domestic partnership
c) In a relationship
d) Single

8. How many children do you have? ___________________

9. Have you ever heard about the term human papillomavirus, better known as HPV?
a) Yes
b) No

10. Have you individually sought information about the HPV vaccine in the Republic of Croatia?
a) Yes
b) No

11. Among the listed sources, please mark all those that were your source of information about the HPV vaccine in the Republic of Croatia:
a) Pediatrician
b) School doctor
c) Other healthcare personnel
d) Pharmacists
e) Media (TV/ radio/ newspapers/ magazines)
f) Forums
g) Internet portals
h) Family members/ friends/ colleagues
i) State institutions (e.g., CHIF, CIPH)
j) Educational institutions (kindergarten/ school/ faculty)
k) I did not use any source nor did I seek information about the HPV vaccine

12. Which of the above sources would you identify as the one you used the MOST frequently?
____

13. In front of you is a series of statements regarding your opinion about the HPV vaccine. Please mark your level of agreement for each statement by placing an "x" next to one number on a scale from 1 to 4, where:

1 - Strongly disagree
2 - Mostly disagree
3 - Mostly agree
4 - Completely agree

	1 - Strongly disagree	2 – disagree	3 - agree	4 – Strongly agree
The HPV vaccine might cause short-term problems, like fever or discomfort.				
The HPV vaccine is being pushed to make money for drug companies.				
The HPV vaccine might cause lasting health problem.				
If a teenage girl/boy gets the HPV vaccine, she/he may be more likely to have sex.				
I think the HPV vaccine is unsafe.				
My child is too young to get a vaccine for a sexually transmitted infection like HPV.				
The HPV vaccine is quite new, so I want to wait a while before deciding if my child should get it.				

14. Would you vaccinate your child against HPV?

a) Yes

b) No

15. Would you leave the HPV vaccination decision up to your child?

a) Yes

b) No

Thank you for your participation!
